# Durable mid‐term survivorship after proximal ACL repair with suture augmentation in a middle‐aged cohort: A risk factor analysis

**DOI:** 10.1002/jeo2.70815

**Published:** 2026-06-22

**Authors:** Anna Patricia Egert, Kristian Nikolaus Schneider, Georg Gosheger, Jan Christoph Theil, Jan Frederic Weller, Alexander Klug, Georg Ahlbäumer

**Affiliations:** ^1^ Department of Orthopaedics and Trauma Surgery Klinik Gut St. Moritz St. Moritz Switzerland; ^2^ Department of Orthopaedics and Trauma Surgery BG Unfallklinik Frankfurt am Main Frankfurt Germany; ^3^ Department of Orthopaedics and Tumor Orthopaedics University Hospital of Münster Münster Germany; ^4^ Department of Hematology and Oncology, including the Pneumology Section University Medical Center Hamburg‐Eppendorf (UKE) Hamburg Germany

**Keywords:** anterior cruciate ligament repair, concomitant injuries, mid‐term follow‐up, risk factors, survival, suture augmentation

## Abstract

**Purpose:**

Arthroscopic primary repair of proximal anterior cruciate ligament (ACL) tears with suture augmentation (SA) provides a ligament‐preserving alternative to reconstruction. Evidence on mid‐term durability and associated risk factors for revision remains limited. This study evaluated revision‐free survival at a minimum follow‐up of 72 months and identified risk factors for revision.

**Methods:**

A consecutive cohort undergoing arthroscopic proximal ACL repair with SA, performed by a single surgeon between 2017 and 2019, was analysed. Revision‐free survival was determined using Kaplan–Meier analysis with 95% confidence interval (CI). Risk factors were evaluated using parametric or non‐parametric tests; significance was set at *p* < 0.05.

**Results:**

A minimum follow‐up of 72 months (median 79, interquartile range [IQR] 75–97) was completed by 80 patients (23 males; mean age 42 ± 13 years). Revision surgery was required in 13 patients (16%) after a median of 38 months, including six re‐ruptures and seven chronic instabilities. Revision‐free survival was 94% at 1 year, 88% at 5 years and 83% at 7 years. Age, body mass index (BMI), sex, surgical timing, meniscal or ligamentous injury and physiotherapy duration showed no significant associations.

**Conclusion:**

Arthroscopic proximal ACL repair with SA demonstrated durable survivorship exceeding 80% at 7 years. While preoperative Tegner score showed an exploratory association with revision risk, no independent predictor of failure was confirmed. These findings support proximal ACL repair with SA as a ligament‐preserving option for carefully selected patients, particularly in middle‐aged individuals with moderate mechanical demand. Generalizability to younger, high‐demand athletes requires further prospective evidence.

**Level of Evidence:**

Level III, retrospective cohort study.

## Graphical abstract

Arthroscopic primary repair of proximal ACL tears with suture augmentation in a middle‐aged cohort (*n* = 80, mean age 42 years) demonstrated a revision‐free survival of 83% at 7 years. Preoperative Tegner score was the only variable showing an exploratory association with revision risk.

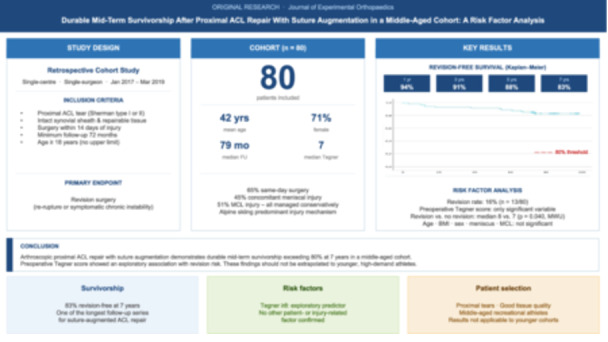

**Abbreviations**
ACL: anterior cruciate ligament
BMI: body mass index
CI: confidence interval
IQR: interquartile range
KM: Kaplan–Meier
LCL: lateral collateral ligament
MCL: medial collateral ligament
MRI: magnetic resonance imaging
PROM: patient‐reported outcome measure
SA: suture augmentation
SD: standard deviation
SPSS: Statistical Package for the Social Sciences

## INTRODUCTION

Primary anterior cruciate ligament (ACL) repair with suture augmentation (SA) has regained interest as a treatment option for proximal ACL ruptures with improved arthroscopic techniques, refined patient selection and modern augmentation techniques [20]. Preserving the native ACL tissue may help to maintain proprioception, avoid donor‐site morbidities and simplify revision surgery if needed due to smaller drill holes [1, 10, 21]. In contrast, ACL reconstruction remains the standard care for the majority of ACL injuries but sacrifices the native ligament and requires graft harvesting [14].

Previous case series of ACL repair with SA have shown promising short‐ and mid‐term outcomes with high patient satisfaction and acceptable failure rates in carefully selected patients [9, 16]. Risk factors for re‐ruptures were reported to be associated with a younger patient's age, higher activity levels of patients as well as sex [6, 15, 17].

However, concerns remain regarding long‐term durability and recurrent instabilities after 5 years, as long‐term data on revision rates and associated risk factors remain scarce [5, 6].

Proximal femoral avulsion tears (Sherman Type I and II) with good tissue quality and an intact synovial sheath appear to provide the greatest healing potential [12, 19, 21]. For these lesions, arthroscopic repair with SA has been proposed as a ligament‐preserving alternative to reconstruction [4, 7]. Yet, the long‐term survivorship of such repairs and the influence of patient‐ and injury‐related factors on failure risk remain unclear.

The purpose of this study was to determine the rate of revision surgery and to identify associated risk factors in a consecutive cohort of patients undergoing arthroscopic proximal ACL repair with SA with a minimum follow‐up of 6 years. It was hypothesized that (1) the technique would demonstrate acceptable mid‐term revision‐free survival in a carefully selected cohort and (2) no specific patient‐ or injury‐related factor would significantly increase the risk of revision.

## METHODS

This retrospective cohort study included all consecutive patients who underwent arthroscopic primary ACL repair with SA at a single tertiary care centre between January 2017 and March 2019.

Ethical approval was received from the local ethics committee, and the study was conducted in accordance with the Declaration of Helsinki. All participants provided informed consent at the time of follow‐up.

### Patient selection

Only patients with an acute ACL rupture with a proximal tear pattern (Sherman Type I or II) with a preserved synovial sheath and repairable tissue quality confirmed intraoperatively with an arthroscope were included. Time from injury to surgery was within 14 days. One patient was included with a treatment delay of 18 days at the patient's request. An age of 18 years or older was required as the minimum inclusion criterion, whereas no upper age limit was applied, as advanced age was not considered a contraindication provided that tissue quality and tear pattern were suitable for repair.

Excluded were patients with a midsubstance or distal ACL tear or poor ACL tissue quality precluding repair, as well as patients who underwent ACL reconstruction.

**Figure 1:**
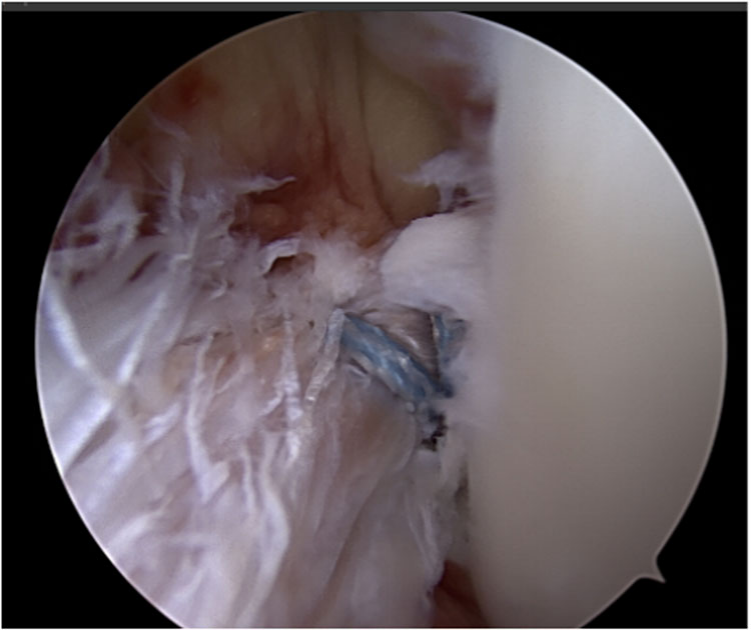
Arthroscopic image of acute ACL re‐rupture 1 year post primary repair with preserved suture augmentation. ACL, anterior cruciate ligament.

Therefore, 80 patients met the inclusion criteria and were eligible for final analysis (Figure 1).

### Surgical technique

The surgical technique has been described in detail previously [15, 16]. Briefly, all procedures were performed arthroscopically by a single senior fellowship‐trained orthopaedic surgeon (G.A.) using a standardized technique. A diagnostic arthroscopy was performed with confirmation of a proximal ACL tear with sufficient tissue quality, including assessment of integrity, fibre alignment and preservation of the synovial sheath.

An SA device (No. 2 FiberWire; Arthrex Inc.) was passed through the remaining ACL tissue approximately 1 cm distal to the rupture site. The femoral tunnel was created using an inside‐out technique at the anatomic femoral footprint with the knee in full flexion, and a shuttle loop was advanced through this tunnel. The tibial tunnel was then drilled in an outside‐in way using an aiming guide positioned at the anterior centre of the native tibial footprint, followed by placement of a second shuttle loop. Both shuttle loops, together with the SA device previously passed through the remnant, were retrieved via the anteromedial portal and guided through the tibial tunnel. The femoral shuttle loop was used to advance the SA construct along with the remnant suture through both tunnels, allowing refixation of the ACL remnant to its anatomic femoral footprint. Proximal fixation was achieved by flipping the button on the femoral cortex, while distal fixation was performed in full extension using a 4.75 mm suture anchor (SwiveLock 4.75 mm, Arthrex Inc.). To enhance biological healing, additional microfracturing was performed at the femoral footprint.

Concomitant meniscal injuries were addressed according to tear pattern and tissue quality. When possible, they were repaired (RapidLoc; Mitek Products) or partially resected if irreparable. Root tears were not present in this cohort. Chondral lesions underwent debridement or microfracturing based on the characteristics of the defect.

Collateral ligament injuries were graded intraoperatively, and all were managed conservatively regardless of grade. No surgical reconstruction of the medial collateral ligament (MCL) or lateral collateral ligament (LCL) was performed in this cohort.

### Postoperative rehabilitation

Postoperative rehabilitation followed a standardized protocol. Patients were allowed partial weight‐bearing with crutches and a hinged knee brace immediately after surgery. Early range of motion was encouraged, with progressive increase in flexion and extension according to pain and swelling. Supervised physiotherapy was conducted by licensed physiotherapists at a frequency of three sessions per week, comprising range‐of‐motion exercises, quadriceps and hamstring strengthening and progressive neuromuscular training. Return to pivoting sports was generally permitted after approximately 6–9 months, based on clinical and functional recovery. The duration of supervised physiotherapy was recorded. A threshold of >12 weeks was applied for analysis, corresponding to the defined end of the intensive supervised phase per protocol.

### Outcome measures

**Figure 2:**
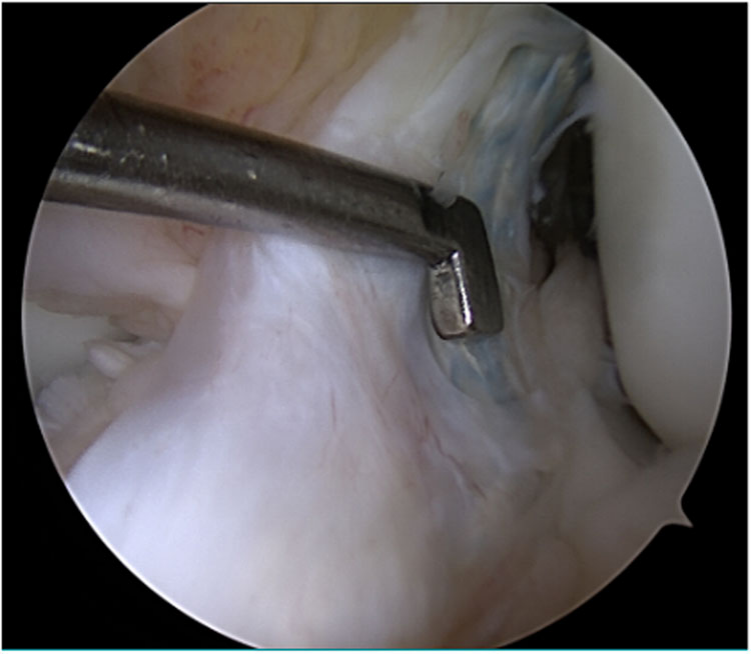
Chronic ACL instability 3 years post‐repair with preserved suture augmentation but elongated native remnant. ACL, anterior cruciate ligament.

The primary endpoint was revision surgery of the ACL‐repaired knee, defined as any subsequent operation addressing ACL failure either due to re‐rupture (Figure 1) or symptomatic chronic instability (Figure 2). The indication and timing of revision surgery were obtained.

Demographic and clinical variables were collected from medical records, including age, sex, body mass index (BMI), timing of surgery (operation on day of injury vs. later, within 14 days) and concomitant meniscal or ligamentous injuries. Concomitant injuries were documented clinically and with magnetic resonance imaging (MRI) prior to surgery and were confirmed intraoperatively. The duration of supervised postoperative physiotherapy (>12 weeks vs. ≤12 weeks) was also assessed via questionnaire.

### Statistical analysis

Statistical analysis was performed using Excel 2024 (Microsoft Corp.) and SPSS 31.0 (IBM Corp.).

Descriptive statistics were used to summarize patient characteristics and outcomes. Continuous variables are presented as mean ± standard deviation (SD) or median (IQR), depending on distribution. Categorical variables are presented as counts and percentages.

Revision‐free survival was analysed using Kaplan–Meier (KM) survival curves with 95% confidence intervals (CIs).

For risk factor analysis, patients who underwent revision surgery were compared with those without revision. Depending on data distribution, independent‐samples *t* tests or Mann–Whitney *U* tests were used for continuous variables and chi‐square tests were used for categorical variables. Data distribution was assessed using the Shapiro–Wilk test. No formal power calculation was performed due to the retrospective design and fixed cohort size. Due to the limited number of revision events, multivariable regression analysis was not performed to avoid model overfitting.

The preoperative Tegner activity score was analysed both as a continuous variable using the Mann–Whitney *U* test (revision vs. no revision) and as a dichotomized variable (threshold ≥8 vs. <8) within the KM framework using the log‐rank test. Both approaches are reported to allow full transparency of the exploratory analysis. Statistical significance was set at *p* < 0.05.

## RESULTS

### Patient characteristics

**Figure 3:**
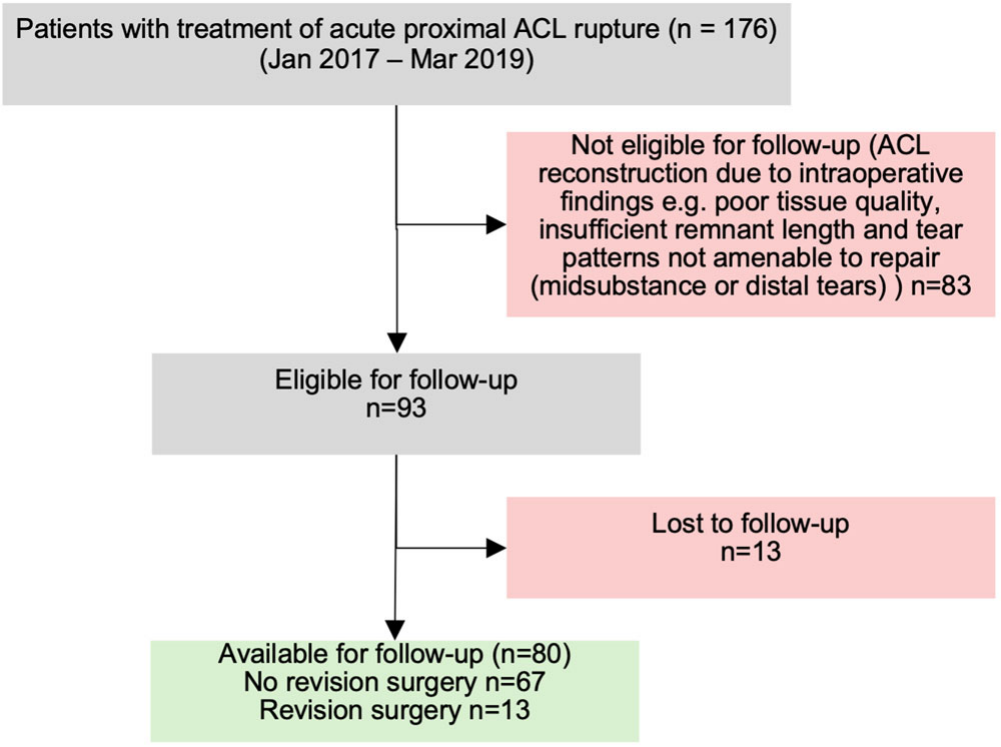
STROBE flowchart of patient selection for this study. Of 176 patients treated for acute proximal ACL rupture between January 2017 and March 2019, 83 were not eligible for follow‐up due to undergoing ACL reconstruction with intraoperative evidence for poor tissue quality, insufficient remnant length and tear patterns not amenable to repair (midsubstance or distal tears). Of the remaining 93 patients eligible for follow‐up, 13 were lost to follow‐up, resulting in 80 patients available for assessment. Among these, 67 patients did not require revision surgery, while 13 underwent revision. ACL, anterior cruciate ligament; STROBE, Strengthening the Reporting of Observational Studies in Epidemiology.

A total of 80 patients (23 males and 57 females) were eligible for follow‐up. The mean age at the time of surgery was 42 ± 13 years. The minimum follow‐up was 72 months, with a median of 79 months (IQR 75–97 months). All patients had proximal ACL tears (Sherman Type I or II), and the majority of patients underwent surgery within 14 days after injury. Due to the patient's wish, one patient was included with a treatment delay of 18 days. Details can be obtained from Table 1 and Figure 3.

**Table 1:** Baseline demographics of the study cohort.

Variable	Total (*n* = 80)
Age at surgery, mean ± SD (years)	42 ± 13.2
Sex—female, *n* (%)	57 (71%)
Sex—male, *n* (%)	23 (29%)
BMI, mean ± SD	24.5 ± 3.9
Preoperative Tegner score, median (IQR)	7 (7–7)
Level <8, *n* (%)	43 (54%)
Level ≥ 8, *n* (%)	12 (15%)
Surgery same day (0–1 days), *n* (%)	52 (65%)
Surgery delayed (2–18 days), *n* (%)	15 (19%)
Follow‐up, mean ± SD (months)	79.0 ± 8.0
Medial meniscus—intact, *n* (%)	44 (55%)
Medial meniscus—meniscectomy, *n* (%)	19 (24%)
Medial meniscus—repair, *n* (%)	4 (5%)
Lateral meniscus—intact, *n* (%)	49 (61%)
Lateral meniscus—meniscectomy, *n* (%)	13 (16%)
Lateral meniscus—repair, *n* (%)	5 (6%)
MCL injury—none, *n* (%)	28 (35%)
MCL injury—Grade I, *n* (%)	17 (21%)
MCL injury—Grade II, *n* (%)	6 (8%)
MCL injury—Grade III, *n* (%)	16 (20%)
LCL injury—none, *n* (%)	43 (54%)
LCL injury—Grade I, *n* (%)	17 (21%)
LCL injury—Grade IV (bony avulsion), *n* (%)	5 (6%)

*Note*: Data are presented as *n* (%) for categorical variables and mean ± SD or median (IQR) for continuous variables. All MCL and LCL injuries were managed conservatively. No surgical ligament reconstruction was performed in this cohort. Meniscectomy includes both isolated partial meniscectomy and bucket‐handle tear with partial resection. Repair includes isolated tear repair and bucket‐handle refixation.

Abbreviations: BMI, body mass index; IQR, interquartile range; LCL, lateral collateral ligament; MCL, medial collateral ligament; SD, standard deviation.

### Revision rate and survivorship

Overall, 13 of 80 patients (16%) underwent revision surgery during the follow‐up period. Revisions were performed after a median of 38 months (IQR 12–61 months) postoperatively. Six patients underwent revision due to traumatic re‐rupture of the repaired ACL and seven patients due to chronic instability without clear traumatic re‐injury.

**Figure 4:**
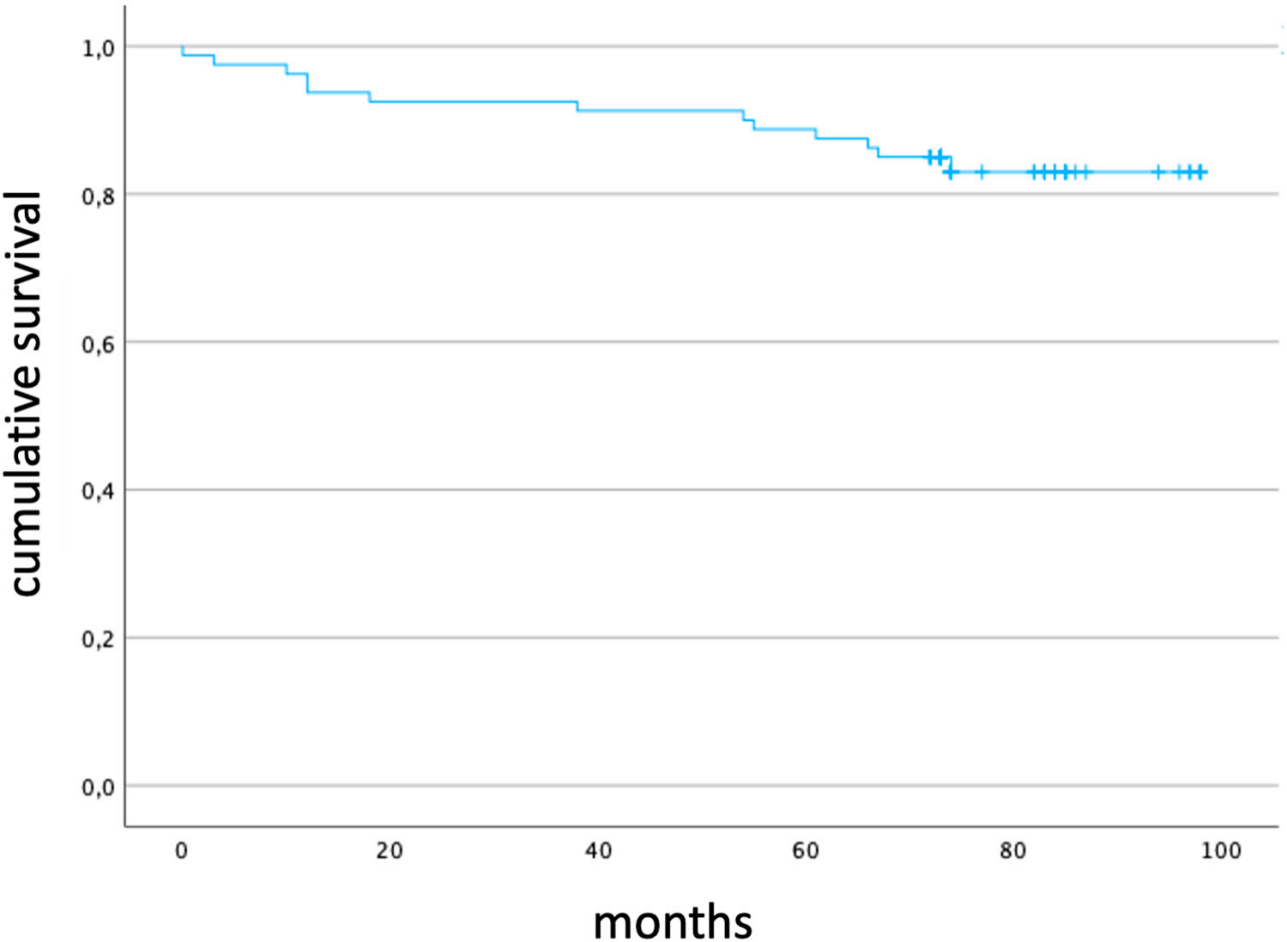
Kaplan–Meier survival curve of arthroscopic anterior cruciate ligament repair with suture augmentation demonstrating a revision‐free survival of 83.0% 7 years after surgery.

KM analysis demonstrated revision‐free survival at 1 year of 94% (95% CI 88–99), 91% (95% CI 85–98) at 3 years, 90% (95% CI 83–97) at 4 years, 88% (95% CI 80–95) at 5 years and 83% (95% CI 74–92) at 7 years after surgery (Figure 4).

### Risk factor analysis

#### Patient‐specific factors affecting survival rate

Among the patient‐specific variables analysed, preoperative Tegner activity score was the only factor to reach statistical significance. Patients who subsequently required revision surgery showed a significantly higher preoperative Tegner score compared with those who did not (median 8 [IQR 7–10] vs. median 7 [IQR 6–7]; *p* = 0.040). In the KM analysis stratified by Tegner score (threshold ≥8), patients with a preoperative Tegner score ≥8 showed a lower 5‐year revision‐free survival compared with those with a score <8, although this difference did not reach significance in the log‐rank test (*p* = 0.156). Age at surgery (*p* = 0.755), sex (*p* = 0.589) and BMI (*p* = 0.586) were not significantly associated with revision risk.

### Injury‐specific factors affecting survival rate

No significant associations were identified between revision surgery and injury‐ or surgery‐related variables, including surgical timing (*p* = 0.954), concomitant medial meniscus injury (*p* = 0.421), lateral meniscus injury (*p* = 0.429), MCL injury (*p* = 0.476), LCL injury (*p* = 0.222) and bony injury (*p* = 0.666).

Likewise, a longer duration of supervised physiotherapy (>12 weeks) was not associated with reduced or increased revision risk (*p* = 0.300).

Details of the named results can be obtained from Table 2.

**Table 2:** Risk factor analysis for revision surgery.

Variable (Group A vs. Group B)	Group A, *n*	5‐year survival A, %, (95% CI)	Group B, *n*	*p* Value
Patient‐specific factors				
Sex (female vs. male)	55	85.5% (76.1–94.8)	25	0.589
Age (≤40 vs. >40 years)	22	81.8% (65.7–97.9)	58	0.755
BMI (≤30 vs. >30 kg/m^2^)	71	85.9% (77.8–94.0)	9	0.586
Tegner score (≥8 vs. <8)	29	96% (90–100.0)	45	0.156 (LR) 0.040* (MWU)
Injury‐ and surgery‐specific factors				
Surgery timing (not same‐day vs. same‐day)	18	83.3% (66.1–100.0)	62	0.954
Medial meniscus injury (no vs. yes)	51	88.2% (79.4–97.1)	29	0.421
Lateral meniscus injury (no vs. yes)	57	87.7% (79.2–96.2)	23	0.429
MCL injury (no vs. yes)	35	82.9% (70.4–95.3)	45	0.476
LCL injury (no vs. yes)	56	82.1% (72.1–92.2)	24	0.222
Bony injury (no vs. yes)	71	84.5% (76.1–92.9)	9	0.666
Physiotherapy ≤12 vs. >12 weeks	22	95.5% (86.7–100.0)	49	0.300

*Note*: Kaplan–Meier estimates at 61 months are reported for continuous group comparisons. Statistical comparisons were performed using the log‐rank) (Mantel–Cox) test for all categorical and dichotomized variables. The Tegner activity score was additionally analysed as a continuous ordinal variable using the MWU test, given its non‐normal distribution and the limited discriminatory power of the ≥8 dichotomization in this cohort. Both results are reported for transparency: MWU test *p *= 0.040; log‐rank test (Tegner ≥8 vs. <8) *p* = 0.156. The Tegner score analysis is highlighted as the only variable reaching statistical significance in the MWU test. Bold values indicate statistical significance (*p* < 0.05).

Abbreviations: BMI, body mass index; CI, confidence interval; LCL, lateral collateral ligament; LR, likelihood ratio; MCL, medial collateral ligament; MWU, Mann–Whitney *U*.

## DISCUSSION

The most important finding of this study was that arthroscopic ACL repair with SA shows a revision rate of 16% at a median follow‐up of 79 months, corresponding to a revision‐free survival of 83% at 7 years in a middle‐aged cohort.

An exploratory finding was the difference in preoperative Tegner activity scores between revision and non‐revision patients (median 8 vs. 7; Mann–Whitney *U*, *p* = 0.040). Given the markedly skewed distribution of Tegner scores in this cohort (median 7, IQR 7–7), the Mann–Whitney *U* test was the appropriate analytic choice. When dichotomized at ≥8 versus <8, the log‐rank test did not reach significance (*p* = 0.156), as this threshold separated only 15 patients from the remainder. It should be noted, however, that the overall low revision rate in this cohort may itself partly reflect the reduced mechanical demand associated with a middle‐aged population with a median Tegner score of 7, rather than representing the intrinsic durability of the technique alone. In this context, the association between higher preoperative activity levels and revision risk is biologically plausible, as greater mechanical demand on the healing ligament may impair biological integration. While prior studies have proposed activity level as a potential failure risk factor, direct evidence using validated preoperative scores remains limited [6, 17]. Given the borderline *p* value, the small number of revision events (*n* = 13) and the skewed score distribution, this finding should be considered hypothesis‐generating. Patients with Tegner ≥8 should nonetheless receive thorough preoperative counselling regarding potentially elevated revision risk.

The mean age of 42 ± 13 years in this cohort is notably higher than in most published series on ACL repair with SA, which predominantly report on patients aged 20–35 years [2, 8, 20]. Older patients typically exhibit lower preoperative activity levels and are less likely to return to high‐demand pivoting sports postoperatively, both of which independently reduce mechanical stress on the healing ligament and may contribute to the favourable survivorship observed in the present cohort. The revision‐free survival of 83% at 7 years should therefore be interpreted with this demographic context in mind and may not be directly comparable to outcomes in younger, more active populations. Generalizability of these findings to high‐demand athletes or patients below 35 years of age cannot be assumed and prospective studies including younger cohorts are needed to define the full scope of appropriate patient selection for proximal ACL repair with SA.

No other patient‐ or injury‐related factor was significantly associated with revision risk.

In the present cohort, the majority of patients underwent surgery on the day of injury, with an injury‐to‐surgery interval of a maximum of 14 days in all standard cases and 18 days in a single case, thereby ensuring that repair was performed in the acute phase with preserved tissue quality. This treatment window is consistent with current concepts of ACL preservation, which recommend early intervention for proximal tears with good‐quality remnants and intact synovial sheath to avoid retraction and histological degeneration of the stump [13, 18]. Previous work has increasingly moved away from considering associated meniscal or chondral lesions as absolute contraindications for ACL repair with SA, provided that the primary indication is fulfilled [4, 6]. While suture‐augmented repair has been shown to yield comparable short‐term functional outcomes to reconstruction, a modestly higher re‐rupture risk has been reported [3]. In line with the present findings, several cohorts have failed to identify meniscal or collateral ligament involvement as independent risk factors for failure after ACL repair with SA and have reported clinically acceptable revision rates despite including patients with combined injuries [2, 6].

The observed revision rate is comparable to or slightly higher than previously reported mid‐term failure rates. This is explained by the substantially longer follow‐up of the present cohort (median 79 months), one of the longest reported for this technique [2, 8, 12, 16, 20]. The extended follow‐up allows detection of late failures not captured in shorter‐term series. Reported revision rates range from approximately 8% to 15% at 2–5 years, depending on cohort characteristics and follow‐up duration [2, 8, 15, 16, 20]. Importantly, most previous studies have reported shorter follow‐up intervals, often around 2 years, whereas the minimum follow‐up in the current series was 6 years, with survivorship still exceeding 80% at 7 years [2, 8, 15, 16, 20]. The findings suggest that, in a carefully selected cohort, durability of suture‐augmented repair extends well beyond the early postoperative phase, with most failures occurring within the first 3 years. This observation suggests that biological healing rather than long‐term mechanical insufficiency may be the primary determinant of successful repair. Comparable results were achieved by Hopper et al. with a revision rate of 17.6% after 5 years of follow‐up [11].

The lack of correlation between age, sex or BMI and revision risk in the present cohort is consistent with several previous reports on ACL repair with SA [2, 14, 20]. While some authors have suggested that very young or highly active patients may be at increased risk of retear after repair, other clinical and experimental studies have indicated that skeletally immature or younger patients may have favourable healing properties and good outcomes after ACL preservation procedures [5, 6]. In the present cohort, even middle‐aged patients (mean age 42 years) achieved stable mid‐term results, suggesting that chronological age alone does not represent a contraindication when tissue quality and tear pattern are suitable.

### Limitations

This study has several limitations that should be acknowledged.

First and most importantly, the cohort has a mean age of 42 ± 13 years, which is substantially higher than most published ACL repair or reconstruction series. The majority of prior studies report on patients in their second or third decade of life, and it remains unclear whether the favourable survivorship observed here would persist in younger, higher‐demand individuals who place greater mechanical loads on the repaired ligament. The findings should therefore be interpreted as specific to a middle‐aged population and cannot be extrapolated to younger athletes without further prospective evidence.

Further, the retrospective design could lead to potential selection bias and limits the ability to establish causal relationships between risk factors and revision outcomes. Although all surgeries were performed by a single experienced fellowship‐trained surgeon, which ensures technical consistency, it also restricts the generalizability of the results to other surgeons or institutions with varying expertise in arthroscopic ACL repair with SA. Outcomes may therefore reflect surgeon expertise and may not be generalizable to lower‐volume settings.

While follow‐up exceeding 6 years is a key strength, functional outcomes such as patient‐reported outcome measures and objective laxity assessment using a knee arthrometer (KT‐1000 or GNRB) were not systematically collected. The absence of arthrometric side‐to‐side difference measurements is a relevant limitation, as it precludes differentiation between patients with subclinical mechanical instability who did not meet the threshold for revision surgery and those who remained truly stable. Consequently, revision‐free survival as the sole primary endpoint may overestimate clinical success and the functional outcome of non‐revision patients cannot be fully characterized.

Furthermore, meniscal injuries were documented by compartment only (medial vs. lateral) and were not further classified by tear type or anatomical location. Lateral meniscus root tears were therefore not recorded as a distinct category; given a reported incidence of 8%–20% in ACL‐injured knees, their presence in this cohort cannot be excluded and represents a limitation of the retrospective data collection.

Patient selection was subject to inherent intraoperative bias, as eligibility for repair was determined arthroscopically based on Sherman classification, tear location and tissue quality, factors that cannot be assessed preoperatively. Consequently, the 83 patients who ultimately underwent reconstruction instead of repair represent a heterogeneous group whose exclusion reasons were not systematically documented, limiting the transparency of the selection process and the generalizability of the present findings.

Another limiting factor is the moderate sample size. Although it represents one of the largest mid‐term cohorts on arthroscopic proximal ACL repair with SA, the number of revision events (*n* = 13) may have reduced the statistical power for detecting smaller effect sizes in risk factor analysis. Additionally, data on patients' postoperative activity levels and return‐to‐sport profiles were not consistently available. Finally, MRI or second‐look arthroscopy was not routinely performed to evaluate graft integrity or biological healing over time. Thus, conclusions about tissue morphology and long‐term remodelling remain unclear.

Furthermore, the duration of supervised physiotherapy, used as a binary variable in the risk factor analysis, may reflect patient compliance rather than a clinical decision, introducing potential confounding that cannot be controlled for in a retrospective design.

Future prospective, multicenter studies with standardized functional testing and imaging follow‐up are needed to validate these findings and refine patient selection criteria.

## CONCLUSION

Arthroscopic primary repair of proximal ACL ruptures with SA demonstrated durable mid‐term survivorship exceeding 80% at 7 years. While preoperative Tegner activity score showed an exploratory association with revision risk, no independent predictor of failure was confirmed. These findings support proximal ACL repair with SA as a reliable ligament‐preserving option for carefully selected patients with acute proximal tears, particularly in middle‐aged individuals with moderate mechanical demand.

## AUTHOR CONTRIBUTIONS

Kristian Nikolaus Schneider, Georg Ahlbäumer, Jan Frederic Weller and Anna Patricia Egert designed the study and collected the data. Kristian Nikolaus Schneider, Georg Ahlbäumer, Jan Christoph Theil and Anna Patricia Egert were responsible for data management, data analysis and preparation of figures. Kristian Nikolaus Schneider, Georg Ahlbäumer and Anna Patricia Egert wrote the manuscript. Jan Christoph Theil, Alexander Klug and Georg Gosheger helped with data analysis and with editing of the manuscript. All authors read and approved the final manuscript.

## FUNDING

The authors have no funding to report.

## CONFLICT OF INTEREST STATEMENT

The authors declare no conflicts of interest.

## ETHICS STATEMENT

This study was approved by Kantonale Ethikkommission Zürich, approval number: 2019‐00758. Informed consent was obtained from all patients.

## Data Availability

The datasets generated and analysed during the current study are available from the corresponding author on reasonable request.
